# Improving the Three-Dimensional Printability of Potato Starch Loaded onto Food Ink

**DOI:** 10.4014/jmb.2311.11040

**Published:** 2024-02-13

**Authors:** Yourim Oh, Seungmin Lee, Nam Keun Lee, Jin-Kyu Rhee

**Affiliations:** Department of Food Science and Biotechnology, Ewha Womans University, Seoul 03760, Republic of Korea

**Keywords:** Jet-type3D printing, potato starch, pea protein, printability

## Abstract

This study focuses on improving the 3D printability of pea protein with the help of food inks designed for jet-type 3D printers. Initially, the food ink base was formulated using nanocellulose-alginate with a gradient of native potato starch and its 3D printability was evaluated. The 3D-printed structures using only candidates for the food ink base formulated with or without potato starch exhibited dimensional accuracy exceeding 95% on both the X and Y axes. However, the accuracy of stacking on the Z-axis was significantly affected by the ink composition. Food ink with 1% potato starch closely matched the CAD design, with an accuracy of approximately 99% on the Z-axis. Potato starch enhanced the stacking of 3D-printed structures by improving the electrostatic repulsion, viscoelasticity, and thixotropic behavior of the food ink base. The 3D printability of pea protein was evaluated using the selected food ink base, showing a 46% improvement in dimensional accuracy on the Z-axis compared to the control group printed with a food ink base lacking potato starch. These findings suggest that starch can serve as an additive support for high-resolution 3D jet-type printing of food ink material.

## Introduction

Meat consumption is expected to increase with the global population and economic progress in developing countries. Increase in meat production causes various problems, including pollution, antibiotic use, spread of infectious diseases, and high resource consumption [1]. One possible solution to these issues involves utilizing vegetable-based raw materials to mimic meat or introducing protein-rich products to the market [2]. Extrusion technology is commonly used in the production of alternative meat [3]. This technology offers the advantage of replicating the texture of real meat by forming protein fibers [4, 5]. Nevertheless, since this method primarily organizes proteins, excluding fat, there are limitations in accurately reproducing the structure of actual meat [6, 7]. To overcome these limitations, the development of alternative meat using other processing technologies is required.

3D printing is a technology suitable for manufacturing customized foods with complex shapes and various textures, and interest in its use as a new food processing technology in the field of food science is increasing [3, 8, 9]. Among the various 3D printing methods, Fused Deposition Modeling (FDM) technology [10], based on an easily accessible extrusion-based printing process, is primarily used for food manufacturing. Notably, 3D-printed products such as chocolate, pasta, cheese, and pizza have been developed using FDM technology [8, 11-13]. However, FDM technology has technical limitations in manufacturing foods with complex and sophisticated structures, such as meat [6].

Inkjet printing (Continuous Ink-Jet (CIJ) or Drop-On-Demand (DOD)) is a technology in the field of bioprinting to manufacture precise cell structures with a resolution of 70 dpi or higher [14, 15]. Although this technology has been sparingly used for 2D image formation of foods like cookies, pizza, and cakes, such as surface decoration and surface injection, inkjet 3D printers are very useful. They can achieve unique microstructures in food by providing complex and precise morphology, along with various textures [8, 16-20].

In food 3D printing, stacking of a high-resolution microstructure is essential for manufacturing high-quality, customized foods [21, 22]. To create 3D-printed foods that accurately reflect customized textures and shapes, high printing precision is crucial [23]. The success of 3D food printing relies on a processing method suitable for extrusion, as precision decreases when layers are under- or over-extruded [6, 24]. In recent years, several methods have been introduced to enhance the printability of intrinsically challenging materials using 3D printing techniques [25]. Among these methods, the addition of flow enhancers such as hydrocolloids, etc. is used to improve printability by providing the viscoelastic properties necessary for extrusion of edible inks [26].

Among hydrocolloid materials, starch plays a crucial role in the food industry as a raw material or food additive owing to its unique functional properties (*e.g.*, cold swelling, water solubility, rheological properties) and relatively low cost [1, 4, 25]. In particular, potato starch, unlike other starches, exhibits a low gelatinization temperature and enhanced water absorption due to the covalently bound phosphate group in amylopectin [27]. These characteristics contribute to stabilizing physical properties and maintaining the shape of the final product [28, 29]. However, the gelatinization of starch can potentially rapidly clog the nozzle. Therefore, the use of native starch is preferred over gelatinized starch, allowing greater modifications in the starch [24, 27, 30-32]. Nevertheless, the use of native starch in food applications has drawbacks, including poor melt processability and low solubility in typical organic solvents. To adapt native starch for food applications, it is often modified through physical, enzymatic, and chemical processes [32].

High shear force technology is commonly used in food applications to disperse solid particles into the continuous phase. By rotating the blade at high speed, this technology facilitates mixing, dispersion, and homogenization, promoting a more uniform distribution in the continuous phase [32, 33]. As starch is subjected to shearing with other ingredients to produce starch-based products, it significantly influences their physical and structural properties. Therefore, analyzing the physicochemical properties of food ink containing potato starch and examining its impact on food quality is crucial.

The primary focus of this study was to produce food ink based on different concentrations of native potato starch through high-speed shearing and to evaluate the characteristics and printability of the ink. As a result, we conducted a detailed study regarding the functional properties of 3D-printed protein products by blending concentrations of potato starch, known for its ability to achieve high resolution, with protein ink.

## Materials and Methods

### Materials

Nanocellulose, alginate, and calcium chloride were purchased from CelluForce (Canada), Yakuri Pure Chemicals Co., Ltd. (Japan), and Samchun Co., Ltd. (Republic of Korea), respectively. Pea protein and potato starch (PS) were purchased from Piowald GmbH (Mühbrook, Germany) and CJ Co., Ltd. (Republic of Korea), respectively.

### Preparation of Food Ink

To evaluate the effect of PS on the resolution of 3D-printed structures, an inkjet 3D printing base ink was prepared using a mixture of nanocellulose-alginate (CA) based on a method proposed by Markstedt *et al*. (2015)[17]. The base ink was prepared by mixing 11% nanocellulose and 2.5% alginate at a 4:1 ratio. Food inks with PS were prepared by homogenizing 20 g of CA with varying proportions of PS (1%, 3%, and 5% w/w) at room temperature. The samples were named according to the materials they contained: CAS for PS, CAP for pea protein, and CAS_1_P for the combination of CAS_1_ and pea protein. Additionally, the nomenclature CAS_n_ is introduced, where 'n' represents the percentage of PS in the food ink base (*e.g.*, CAS_1_ for 1% PS).

### Zeta Potential and Refractive Index

The zeta potential and particle size of the food inks were evaluated using Otsuka Electronics (ELSZ-2000, Japan) with a refractive index of 1.33. Samples were diluted before measurement, and all analyses were conducted at 25°C.

### Morphology

Morphology was assessed using a low-vacuum scanning electron microscope (SEM) (TM3030Plus tabletop microscope; Hitachi High-Technologies, Japan) after freeze-drying the samples. SEM analysis was performed at EDX to obtain images at 600× magnification with a scattering and backscattering electron signal. The distance between granules was analyzed using the open-source software ImageJ (National Institutes of Health, USA).

### FTIR Spectroscopy

FT-IR spectra were analyzed using a spectrometer (Cary 630 FTIR, Agilent, USA) within the wavenumber range of 4,000-650 cm^-1^, with a resolution of 4 cm^-1^ and 8 scans.

### Measurement of Rheological Properties

The dynamic viscoelastic properties of the food inks were measured using a rheometer (MCR102, Anton Paar, Austria) with a 25 mm plate-plate geometry and a 1 mm gap between the plates. Measurements were performed at 25°C, and a strain sweep at 1 Hz was applied to determine the linear viscoelastic section. The dynamic viscoelastic properties were assessed by angular frequency sweep within the range of 0.1 to 100 rad/s. The complex viscosity (η*) and loss factor (tan δ) were recorded using an Anton Paar RheoCompass 1.22 system.

A Three Interval Thixotropy Test (3ITT) was applied to characterize the complex viscosity recoverability of the food inks using a low shear strain of 1% for 30 s, followed by a high shear strain at 10% for 30 s, and finally a low shear strain at 1% for 30 s. The complex viscosity recoverability of the inks was determined as the percentage of complex viscosity at 8 s, 16 s, 24 s, and 32 s in the third step after the end point based on the complex viscosity in the first step.

Complex viscosity recoverability (η*_rec_, %) = (End point complex viscosity of first interval/ Recovered complex viscosity of third interval) × 100

### Process of 3D Printing

A 3D Inkjet Printer equipped with a micro-dispenser from Vermes (MDC 2090A, Germany) was used for constructing food 3D structures (Fig. S1). The printer head, composed of micro valves based on piezo-electric jet technology, included two heads (CAS head and CaCl_2_ head) for cross-linking. The flow rate was controlled through the rising (valve rise), open time (valve open), falling (valve closes and pressure is applied to the liquid remaining in the nozzle compression chamber), delay (adjusted value and interruption of the internal system process), and needle lift (adjusting the range of the tappet). The printing parameters are detailed in Table S1. A CAD design was generated using Solidworks (Dassault Systemes, France) and was saved in *.STL file format (Fig. S1B). The file was uploaded to 3D slicing software (Simplify 3D 4.1.1, Simplify 3D, USA), and the resulting *G-code file was applied to the 3D printer to execute printing with food ink.

### Evaluation of Accuracy

Dimensional accuracy between the CAD design and 3D structure (honeycombs, 20 × 20 × 10 mm) was evaluated using a vernier caliper (Digital Caliper, Japan). The accuracy was determined by comparing the measured dimensions of the X-axis, Y-axis, and Z-axis to the CAD-designed dimensions using the following formula.

Accuracy = (3D structure length-CAD design length)/CAD design length × 100

### Statistical Analysis

All experiments were conducted at least in triplicate, and results are presented as mean ± standard deviation. Duncan’s multiple range test and one-way analysis of variance (ANOVA) was used for statistical analysis using SPSS (SPSS ver. 12.0, SPSS Inc., USA). The significance level was *p* < 0.05.

## Results and Discussion

### 3D Printability of Inkjet Food Ink with Potato Starch

**Fig. 1:**
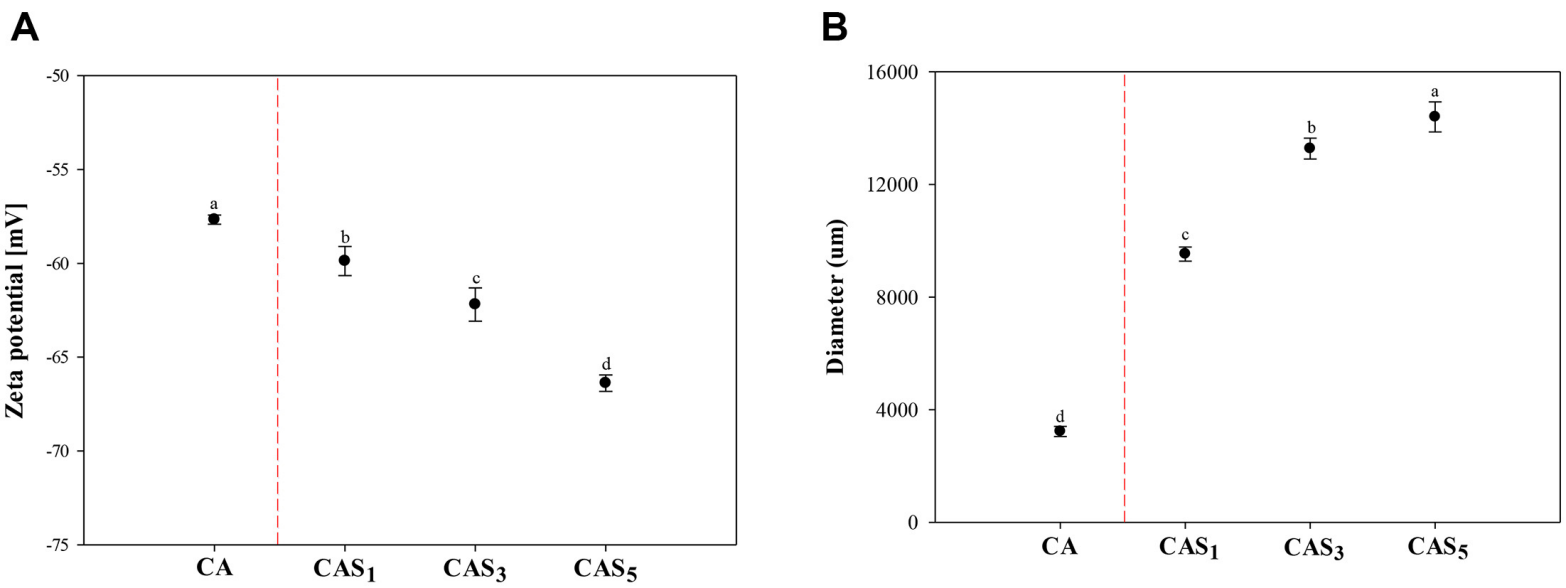
Influence of the concentration of native PS on the zeta potential and hydrodynamic diameter. (A) Zeta potential; B, Hydrodynamic diameter. CA, ink without PS; CAS_1_, CA with 1% PS; CAS_3_, CA with 3% PS; CAS_5_, CA with 5% PS.

Zeta potential is commonly used to estimate exposure to ionic charges and to evaluate colloidal stability through electrostatic repulsion, reflecting the interaction energy between particles based on DLVO theory [34]. Additionally, the zeta potential serves as a crucial criterion for electrostatic repulsion, predicting the potential stability of hydrocolloid systems and aiding in obtaining stable formulations for production [35]. Electrostatic interactions between identical ionic charges on particles repel each other, contributing to dispersion stability [36]. In general, dispersion systems are considered stable when the zeta potential is higher than +30 mV or lower than -30 mV [37]. Fig. 1A and 1B display the zeta potential and hydrodynamic diameter of the food inks, respectively. The zeta potential of the food inks was less than -30 mV, indicating homogenization through high-speed shear and stable dispersion (Fig. 1A). The CA was confirmed to carry a negative charge, attributed to the composition of the base ink, which includes sodium alginate consisting of (1, 4)-linked β-D-mannuronic and α-L-guluronate units [38]. Additionally, nanocellulose is characterized by sulfate groups resulting from hydroxyl esterification [39]. Both sodium alginate and nanocellulose are anionic in nature. As observed in CAS, the inclusion of PS in CA ink resulted in a lower zeta potential than CA ink without PS, enhancing the stability of granules in ink dispersion. Granules of PS characterized by large size, smooth texture, and long amylopectin chains containing high -molecular-weight amylose [40] contribute to this effect. In particular, phosphate monoesters covalently bound to amylopectin possess a negative charge, causing them to attract cations and repel anions [41]. As a result, CA, characterized as a negatively charged, enhanced electrostatic repulsion between PS granules that facilitates the formation of a percolated network structure, led to an increased zeta potential [35]. Fig. 1B shows the mean hydrodynamic diameter corresponding to 90% of the dispersion. The diameter of food inks increased with the concentration of PS. The Polydispersity Index (PdI) characterizes the size range in relation to the distribution of particle size, with a PdI between 0.1 and 0.7 indicating a nearly monodisperse sample [42, 43]. The PdI values of CA and CAS_1_ were approximately 0.37 and 0.66, respectively, suggesting nearly monodisperse distributions. However, CAS_3_ and CAS_5_ exceeded 0.7 as the PS content increased, indicating a broader particle size distribution (data not shown).

**Fig. 2:**
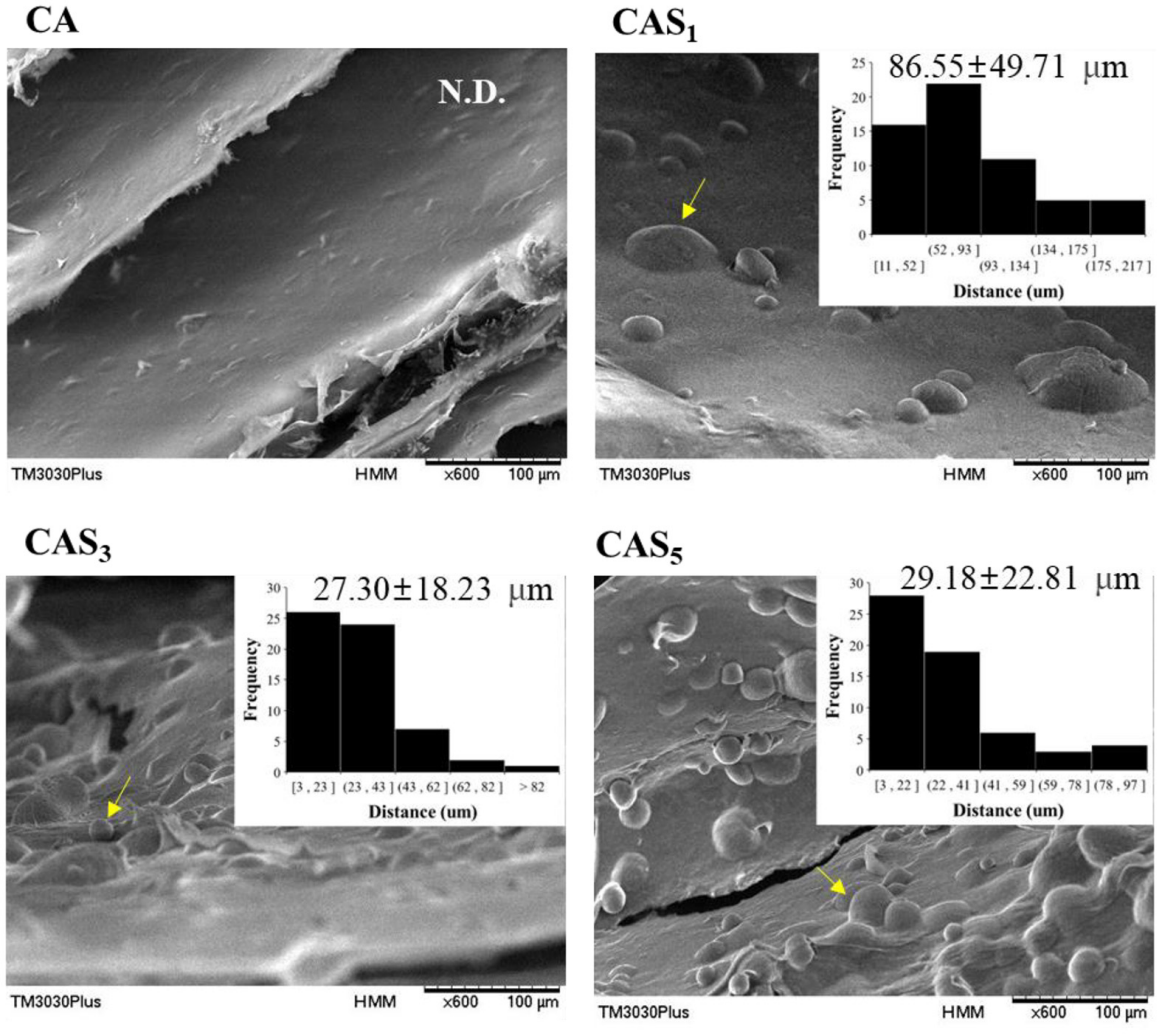
SEM micrographs of the surface and granule distance of food inks. CA, ink without PS; CAS_1_, CA with 1% PS; CAS_3_, CA with 3% PS; CAS_5_, CA with 5% PS. Arrow, PS granules with an oval shape.

The microstructure of the food ink is depicted in Fig. 2, and a smooth CA surface was observed. The raw materials used in CAS_n_ ink were all negatively charged, and the robust electrostatic repulsion between these negative charges caused the PS to adhere to the surface without penetrating the CA. Starch granules exhibited minimal structural changes due to high-speed shearing. This observation is consistent with previous results from Hidalgo-Tufiño *et al*. that suggest preservation of the majority of the initial granular structure despite some alterations of the starch surface by high-speed shearing [33]. Furthermore, with an increase in the concentration of PS, the quantity of starch adhering to the surface of CA continued to increase, resulted in entanglement due to cross-linking between granules [44]. Consequently, the distances between starch granules in CAS_3_ and CAS_5_ became approximately 68% and 66% closer than in CAS_1_, respectively.

**Fig. 3:**
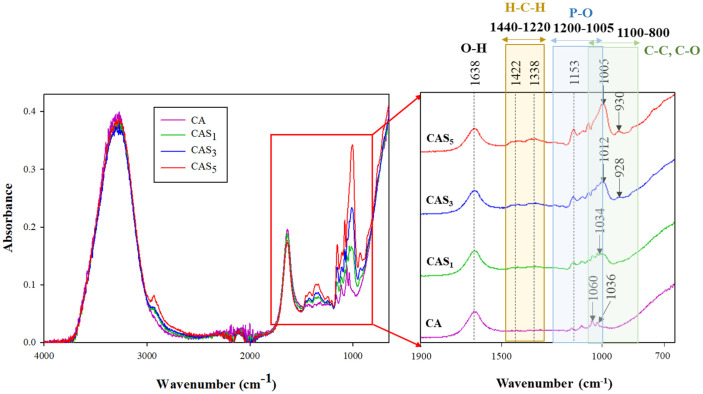
FTIR spectra of food inks according to concentration of native PS. CA, ink without PS; CAS_1_, CA with 1% PS; CAS_3_, CA with 3% PS; CAS_5_, CA with 5% PS.

The functional groups and chemical bonds of the food inks were characterized using FTIR spectra (Fig. 3). With an increase the concentration of PS, new functional groups were generated, and an interaction occurred between CA and PS. The primary bands observed in the food ink were in the regions of 1100-800 cm^-1^, 1200-1005 cm^-1^, 1440-1220 cm^-1^, and 1638 cm^-1^. The 1100-800 cm^-1^ range corresponds to C-C and C-O stretching [41], 1200-1005 cm^-1^ represents the stretching bands of the phosphate group [45], 1440-1220 cm^-1^ involves the bending of the H-C-H bond [46], and 1638 cm^-1^ signifies the H-O-H bending vibration [47]. The 1200-1005 cm^-1^ region, originating from PS, denotes asymmetric stretching vibrations of the P-O bond as well as H-O-H, C-O-O, and C-OH bonds (the presence of hydroxyl groups) [48]. With the addition of PS, the intensity of the band at 1638 cm^-1^ decreased, while that of peaks in the 1100~800 cm^-1^ and 1200~1005 cm^-1^ regions increased, and a new band at 1440-1220 cm^-1^ emerged.

**Fig. 4:**
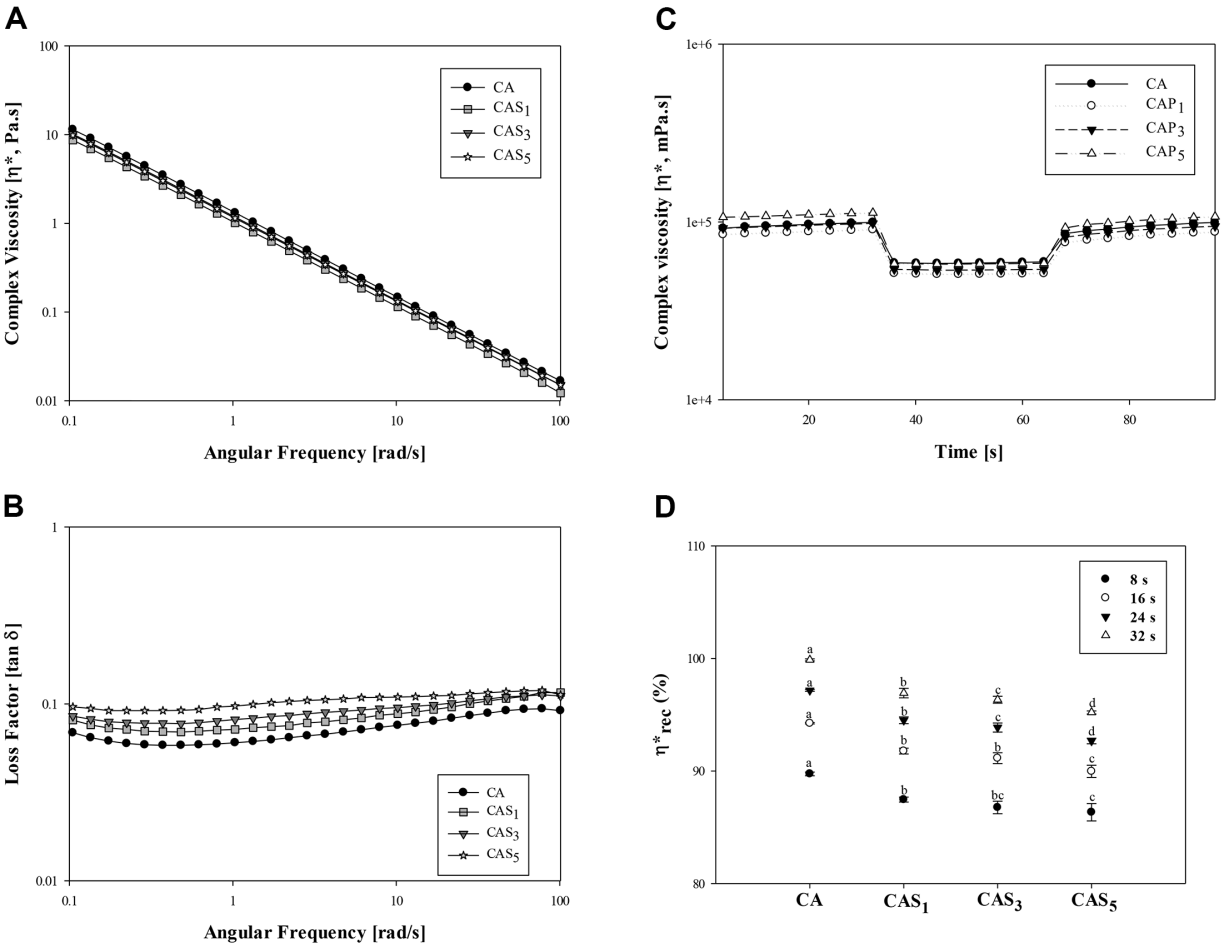
Rheological properties of inks with addition of different concentrations of native PS. (**A**) Complex viscosity (η*) according to the frequency sweep test; (**B**) Loss Factor (tan δ) according to the frequency sweep test; C, thixotropic behavior; D, percentage viscosity recovery as a function of time. CA, ink without PS; CAS_1_, CA with 1% PS; CAS_3_, CA with 3% PS; CAS_5_, CA with 5% PS.

In general, food ink ideal for 3D printing needs to exhibit optimal shear-thinning behavior to endow flowability and appropriate thixotropy to induce buildability of the printed structure [49]. In order to meet these requirements, the ink should behave as a fluid during 3D printing and display elastic behavior at rest and when no stress is applied [50]. To simulate the flow and viscoelastic properties of food ink during 3D printing, frequency sweep tests and three interval thixotropy (3ITT) analyses were performed. The complex viscosity (η*) was measured to assess the flowability of the ink, where η* decreased as the frequency increased, confirming shear-thinning behavior conducive to extrusion (Fig. 4A). The loss factor (tan δ), representing the ratio of the loss modulus (G") to the storage modulus (G'), was used to evaluate the viscoelasticity of the sample. Generally, a value less than 1 indicates elastic behavior (solid-like), while a value exceeding 1 indicates viscous behavior (liquid-like)[51]. All food inks exhibited values less than 1, signifying elastic behavior. However, as the concentration of PS was increased, viscosity decreased (Fig. 4B). This corresponds with the results indicating low viscosity resulting from the robust electrostatic interaction between PS granules and the negatively charged xanthan gum [36]. To mimic the shear conditions of 3D printing, a low shear strain of 1% was initially applied to the ink to simulate the initial shear force in the syringe. Subsequently, during extrusion, the shear strain was increased to 10% to simulate the shear force at the nozzle tip. To simulate the event after 3D printing, the shear force was reduced back to 1% to test the recovery behavior of the food ink. The viscosity of the food ink was responsive to varying shear forces and changes over time, as Fig. 4C and 4D. The shear recovery behavior across a range of shear forces is shown in Fig. 4C, whereas the viscosity change over time is shown in Fig. 4D. The viscosity of all food inks decreases at high shear forces and recovers at low shear forces (Fig. 4C). While the viscosity of CA ink quickly reached a stabilized state within 32 s, an increase in PS concentration resulted in longer sample reconstruction times, indicating higher thixotropic behavior (Fig. 4D). This heightened thixotropy was attributed to the delayed recovery of the percolated structure after shearing of the starch granules [35].

**Fig. 5:**
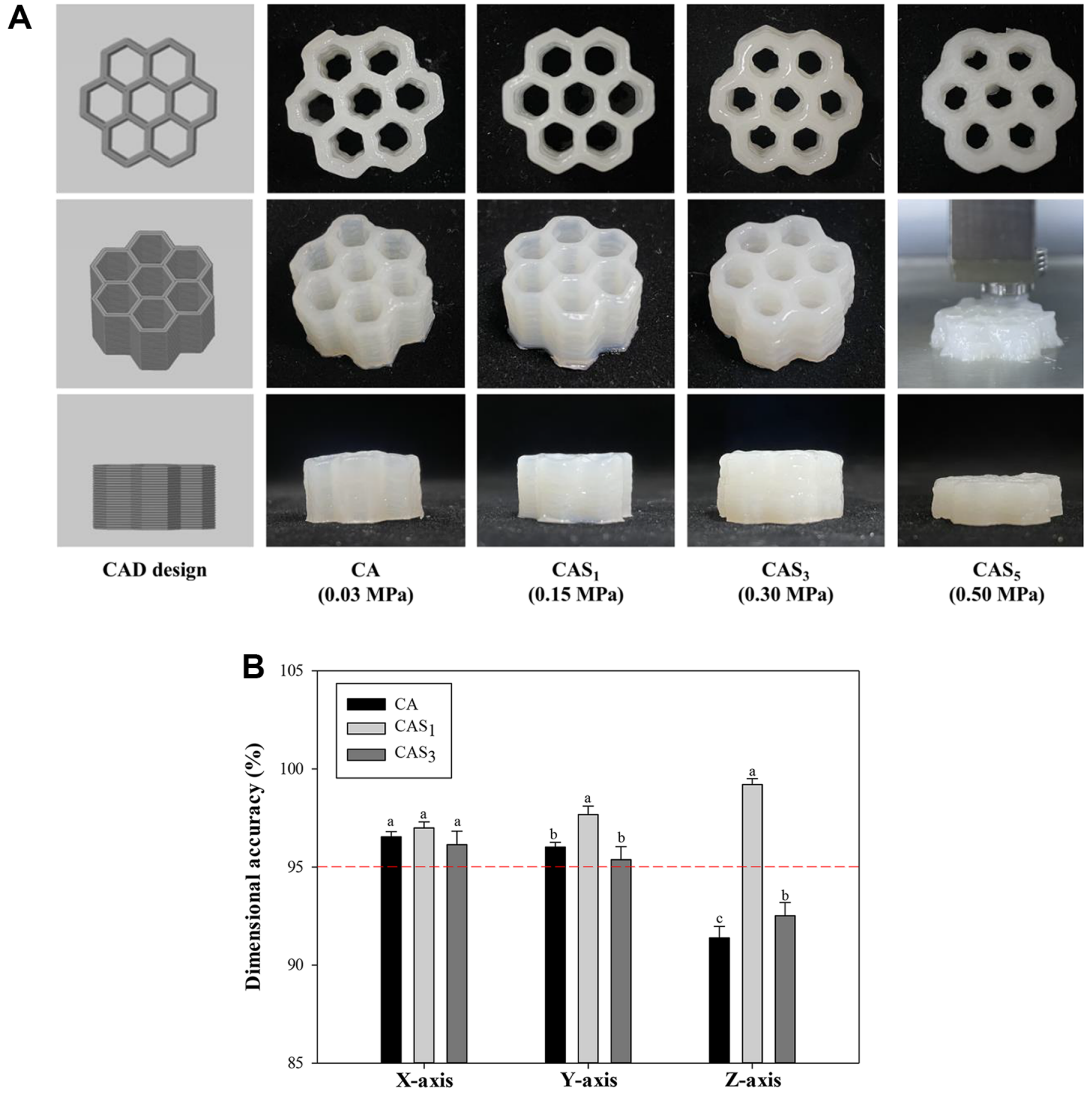
Printability evaluation of a 3D-printed structure. (**A**) Morphology; (**B**) Error of accuracy of 3D structure dimensions. CA, structure without PS; CAS_1_, CA with 1% PS; CAS_3_, CA with 3% PS; CAS_5_, CA with 5% PS. The different letters indicate that the mean values were significantly different (*p* < 0.05).

The geometrical integrity of printed structures is significantly influenced by the nozzle, which is one of the most important components in a 3D printer [52]. A smaller nozzle in 3D printing might enhance dimensional resolution and surface quality but requires a higher extrusion pressure. Insufficient pressure may lead to nozzle clogging, and the 0.4 mm nozzle used in this experiment is considered small according to Liu *et al*. (2019) [53]. Additionally, the extrusion pressure is a determining factor for the flowability of 3D printed material. Insufficient pressure can result in an uneven pattern when the food ink is expelled from the nozzle, while excessive pressure may lead to build-up problems and poor resolution [30]. Under the conditions outlined in Table S1, the minimum pressure required for extrusion from the same nozzle differed by ink (Fig. 5A). The pressure applied during 3D printing increased with higher starch concentration. Consequently, 3D-printed structures with CA exhibited rough surfaces and uneven shapes (Fig. 5A), likely due to the elastic behavior of CA (Fig. 4B), which hindered smooth flow when the food ink was extruded under applied pressure. This issue was alleviated by the addition of PS. However, with an increase in the starch concentration, the applied pressure on the food ink increased, ultimately leading to stacking and resolution problems. In the CAS_5_ ink, characterized by significant PS entanglement, a pressure of 0.5 MPa was applied, leading to an augmented extrusion volume [54]. As a result, the contact of the nozzle during ink extrusion needs to be considered more carefully. Therefore, dimensional accuracy was measured immediately after 3D printing with CA, CAS_1_, and CAS_3_ structures that had undergone the 3D printing process (Fig. 5B). The seamless 3D printing of food underwent quality control through a printability assessment and dimensional accuracy evaluation employing a crucial validation method [55]. He *et al*. (2019) reported that 3D printing accuracy and shape stability were achieved when the accuracy exceeded 95% [56]. The dimensional accuracy of the 3D structure along the X and Y axes consistently exceeded 95%, while the accuracy on the Z-axis exhibited notable variations depending on the starch concentration (Fig. 5B). With CA, rapid structure reconstruction with robust Z-axis recovery (Fig. 4D) resulted in decreased accuracy as the layer thickness increased. CAS_1_, characterized by high thixotropy (Fig. 4D), improved ink flow and demonstrated the most stable Z-axis shape with 95% dimensional accuracy. These findings suggest that CAS ink components, all of which are negatively charged, induced strong electrostatic interaction, reducing viscosity and enabling the Z-axis to be 3D-printed similarly to the CAD design. CAS_3_, with higher viscosity, required a higher pressure due to starch entanglement as the concentration increases, leading to elevated extrusion volume and reduced accuracy.

### 3D Printing of Potato Starch-Containing Pea Protein Ink

**Fig. 6:**
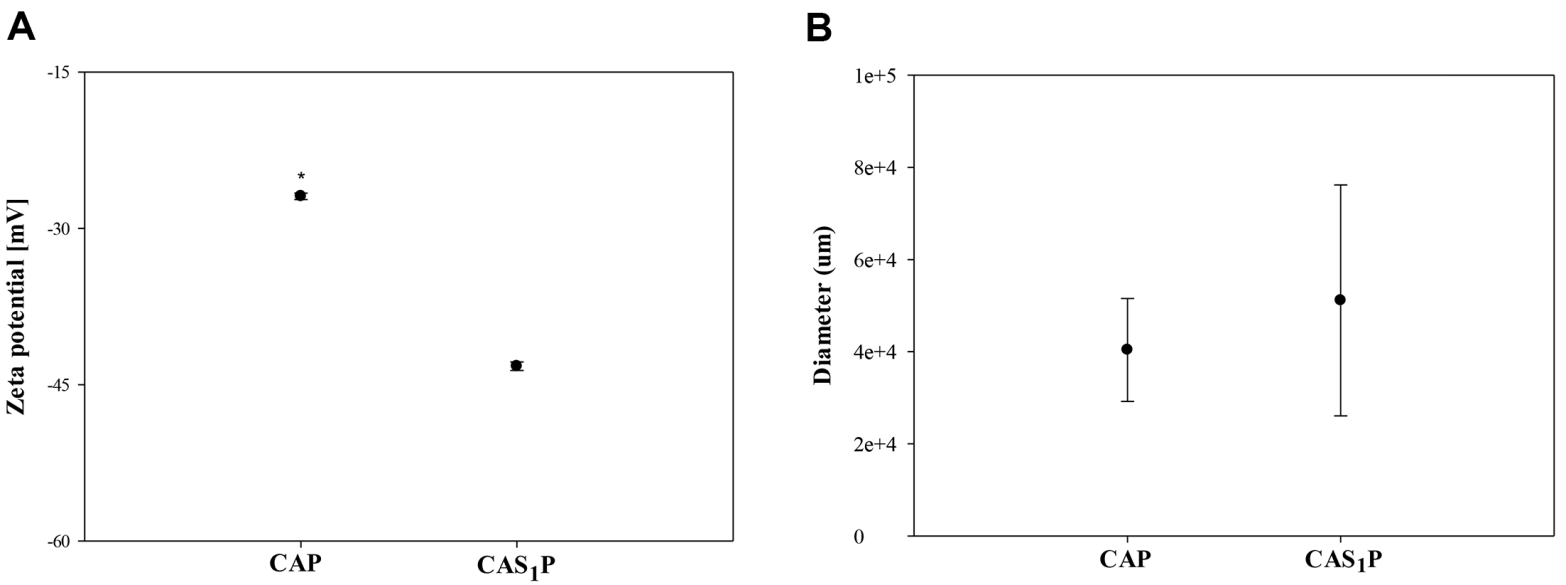
Influence of the addition of native PS on the zeta potential and hydrodynamic diameter. (**A**) Zeta potential; (**B**) Hydrodynamic diameter. CAP, protein ink without PS; CAS_1_P, protein ink with 1% PS.

The dimensional accuracies on the X and Y axes in the 3D-printed structures of CAP were high, reaching 96%, while the accuracy of the Z-axis was notably lower at 68% (Fig. 10). To improve the printing accuracy of 3D structures produced with CAP ink, the printing accuracy was investigated by adding 1% PS to pea protein ink (CAS_1_P) based on the results of the CAS_1_ experiment. The CAP ink was prepared by blending 3% pea protein powder and CA, and the zeta potential and hydrodynamic diameter of each were measured. The zeta potential of CA ink was -58 mV (Fig. 1A); when loaded with 3% pea protein, the negative charge was weakened to -27 mV (Fig. 6A). The zeta potential serves not only to gauge dispersion stability through particle repulsion, but also to assess the strength of attraction. A lower absolute value indicates a stronger attractive than repulsive force when two particles share the same charge [57]. Various molecular interactions occur between proteins and polysaccharides, influencing the functional performance of edible inks [58]. The zeta potential of CAP ink was less negative than that of CA without pea protein. This is likely due to the electrostatic attraction between the negatively charged CA and the positively charged amino acids (lysine and arginine) abundant in pea protein [59, 60].

The zeta potential of the CAS_1_P ink was -43 mV. The increase in negative charge of CAS_1_P increases the electrostatic repulsion between CAP and PS granules, leading to more efficient formation of a percolated network structure than with CA. This efficiency can reduce the aggregation of granules [35]. The hydrodynamic diameter of granules in CAS_1_P increased with the load of PS (Fig. 6B). The PdI values were 1.03 and 1.21 for CAP and CAS_1_P, respectively, and entanglement and polydispersity were observed in all samples [43]. This is consistent with the findings from CAS_3_ and CAS_5_, which showed entanglement and polydispersity (data not shown).

**Fig. 7:**
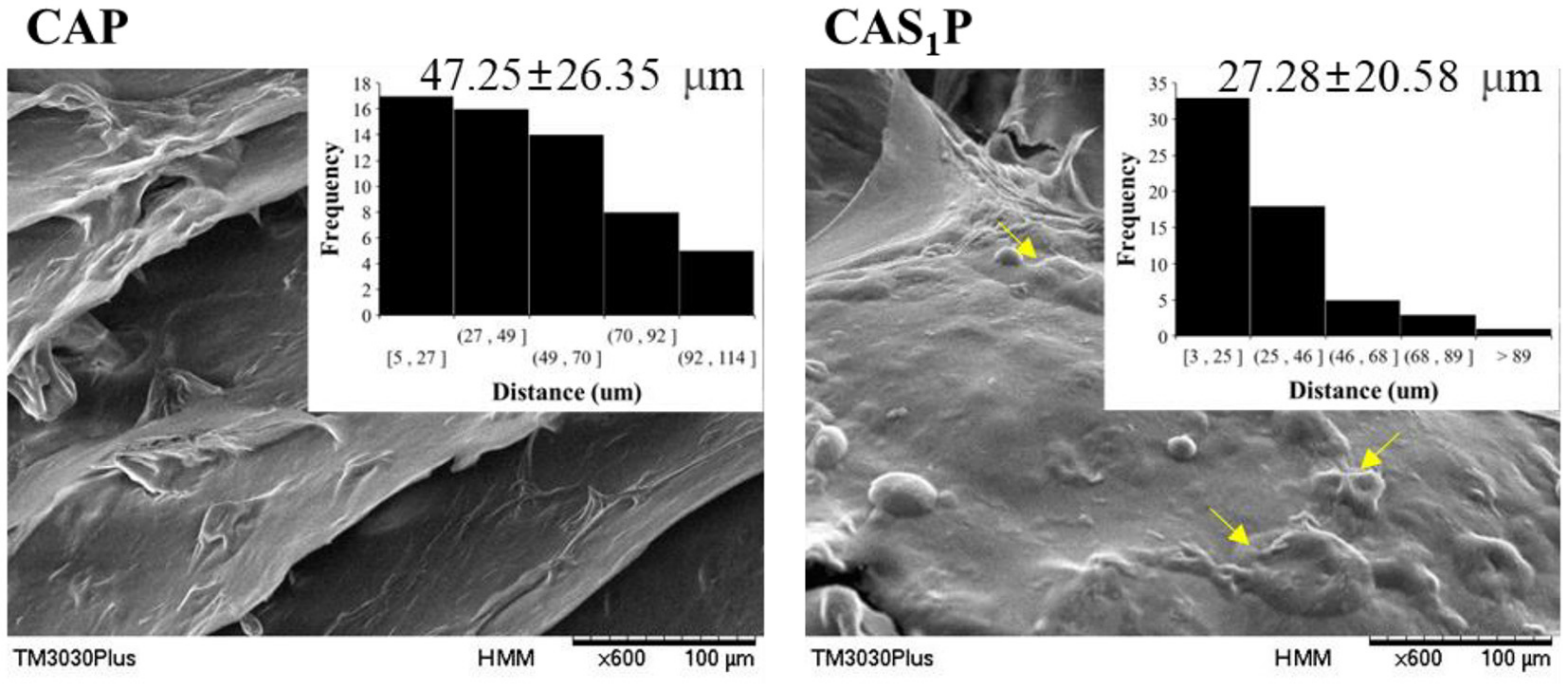
SEM micrographs of the surface and granule distance of food inks. CAP, protein ink without PS; CAS_1_P, protein ink with 1% PS. Arrows, intergranular cross-linking entanglement of pea protein and PS.

Regarding microstructures CAS_1_P had a rougher surface than CAP due to entanglement between pea protein and PS granules (Fig. 7). Such entanglement led to a 42% shorter distance between granules than in CAP.

**Fig. 8:**
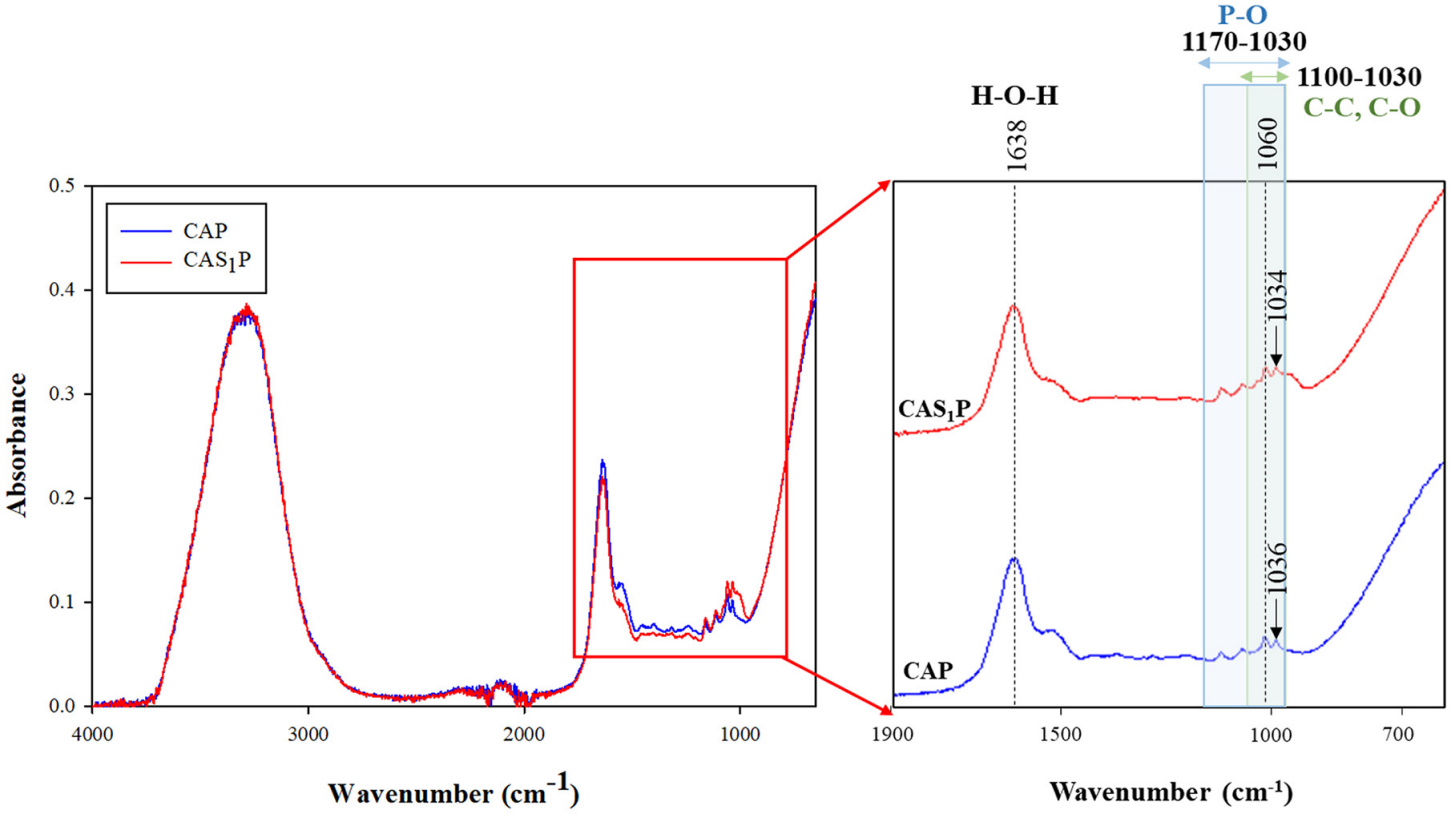
FTIR spectra of food inks according to native PS. CAP, protein ink without PS; CAS_1_P, protein ink with 1% PS.

In the FTIR analysis conducted to identify functional groups and chemical bonds of CAP and CAS_1_P inks, the prominent bands observed in the inks were in the ranges of 1100-800 cm^-1^, 1200-1005 cm^-1^, and 1638 cm^-1^. Similar to the results of the CA ink depending on the concentration of PS (Fig. 3), the intensity of the peak at 1060 cm^-1^ increased and the intensity of the peak at 1638 cm^-1^ decreased (Fig. 8).

**Fig. 9:**
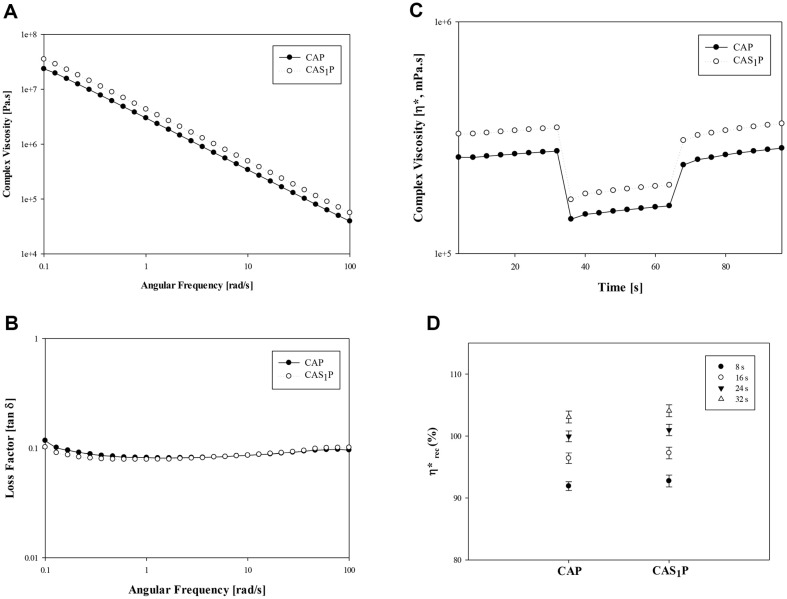
Rheological properties of inks with addition of different concentrations of native PS. (**A**) Complex viscosity (η*) according to the frequency sweep test; B, Loss Factor (tan δ) according to the frequency sweep test; C, thixotropic behavior; D, percentage viscosity recovery as a function of time. CAP, protein ink without PS; CAS_1_P, protein ink with 1% PS.

CAP and CAS_1_P inks were exhibited shear-thinning behavior, with η* decreasing as the angular frequency increased (Fig. 9A). The tan δ values for CAP and CAS_1_P inks were both smaller than 1, indicating excellent elastic behavior (Fig. 9B). Both inks demonstrated a decrease in viscosity at high shear force (shear strain of 10%), consistent with the results shown in Fig. 4C, and it recovered at low shear force (shear strain of 1%) (Fig. 9C).

CAP and CAS_1_P inks showed a difference in viscosity in areas with high shear force (shear strain of 10%). Various studies have shown that proteins, either alone or in combination with polysaccharides such as native starch or inulin can form gel matrices with different microstructures, enhancing rheological properties [52]. This suggests that the addition of PS to CAP ink change the microstructure of the gel matrix, improving the viscosity of the ink.

**Fig. 10:**
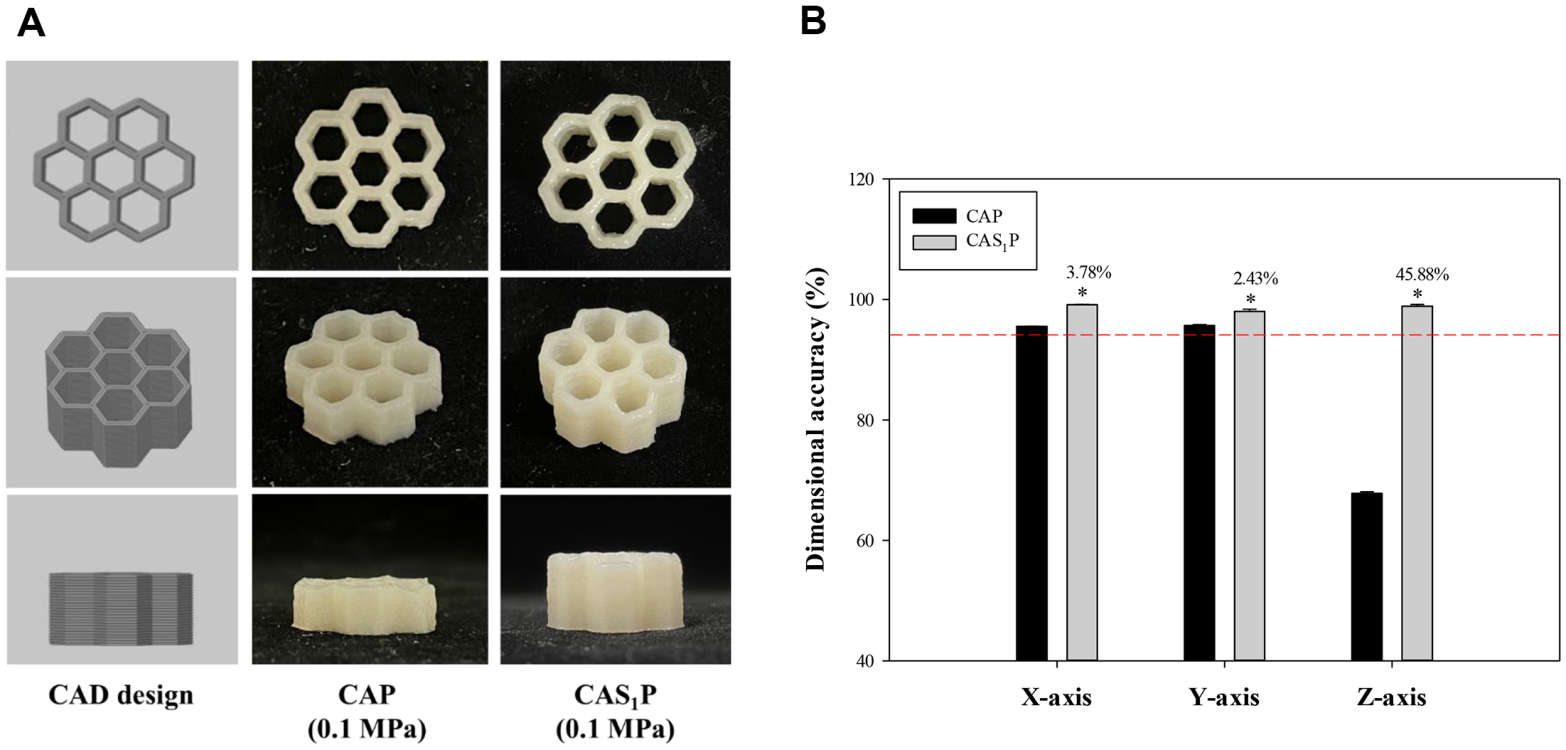
Printability evaluation of the 3D-printed structure. (**A**) Morphology; B, Error of accuracy of 3D structure dimensions. CAP, protein ink without PS; CAS_1_P, protein ink with 1% PS. The different letters indicate that the mean values were significantly different (*p* < 0.05).

The viscosity changes of CAP and CAS_1_P inks over time stabilized within 16 s to 24 s for both inks, with CAS_1_P showing a faster recovery rate (Fig. 9D). Both inks can be extruded at the same pressure of 0.1 MPa, and the stacked 3D structures had a dimensional accuracy greater than 95% in both the X and Y axes (Fig. 10). In the Z-axis, the structure of CAP had a low resolution of about 68%, but that of CAS_1_P improved by about 46% with the addition of PS (Fig. 10B).

When the pea protein content exceeds 2%, the degrees of cross-linking and cohesion decrease, leading to deterioration of the shape and resolution of 3D-printed structures [61]. As CAP contained 3% pea protein content, the resolution was poor due to reduced cohesion. However, the resolution of CAS_1_P was improved because of the interactions between PS granules and the pea protein matrix.

## Conclusion

This study elucidates that the preparation of food ink through high-speed shearing without gelatinizing natural PS significantly influenced the functional properties of the ink. Native PS contributed to increased dispersion stability through electrostatic repulsion at the same charge and played a pivotal role in the flowability of the ink. In contrast, CAP ink, with different charges between CA and pea protein, allowed a lower extrusion volume and resolution due to electrostatic attraction. These findings highlight that the rheological properties and resolution were enhanced by combining the polysaccharides of PS and pea protein, creating a gel matrix with a distinct microstructure. Since food ink comprises various ingredients, the material properties can impact printability. PS emerged as a material capable of improving resolution by interacting with the composed materials, leading to electrostatic repulsion and the formation of a new gel matrix. Therefore, PS provides valuable insights for production of 3D-printed foods with high resolution.

## Supplemental Materials

Supplementary data for this paper are available on-line only at http://jmb.or.kr.
