# Supplementary material for: Improving the Three-Dimensional Printability of Potato Starch Loaded onto Food Ink
Source: J Microbiol Biotechnol. 2024 Feb 13;34(4):891–901. doi: 10.4014/jmb.2311.11040 (PMC11091668; doi:10.4014/jmb.2311.11040)
Supplement: Supplementary file 1 [file jmb-34-4-891-supple.pdf]

J. Microbiol. Biotechnol.
https://doi.org/10.4014/jmb.2311.11040

Supplementary Figure and Table

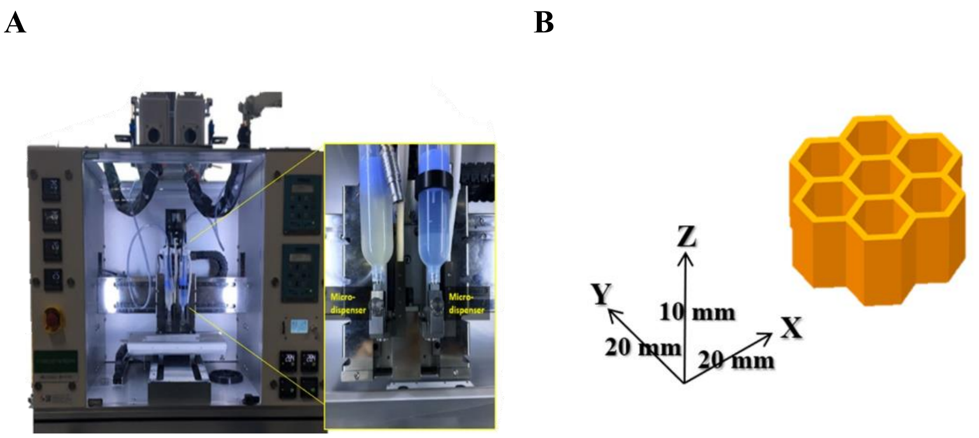

Fig. S1. 3D inkjet printer and CAD design. **(A)** Dual micro-dispenser; **(B)** CAD design for 3D printing.

Table S1. Parameters of 3D printing.

Parameter		$CaCl_2$ Head	CAP Head
Simplify 3D software	Nozzle diameter (mm)	0.4	0.4
	Print speed (mm/min)	650.0	650.0
	Layer height (mm)	0.33	0.33
	First layer height (%)	100	130
	Extruder temperature (℃)	25.0	25.0
	Nozzle temperature (℃)	25.0	25.0
	Bed temperature (℃)	25.0	25.0
3D printer	Pressure (MPa)	0.0	0.03-0.50
	Rising (ms)	7.0	0.5
	Open (ms)	0.3	0.2
	Falling (ms)	1.6	0.7
	Delay (ms)	40.0	42.3
	Needle (%)	100.0	100.0
